# Supplementary material for: Regional and age-dependent changes in ubiquitination in cellular and mouse models of spinocerebellar ataxia type 3
Source: Front Mol Neurosci. 2023 Apr 14;16:1154203. doi: 10.3389/fnmol.2023.1154203 (PMC10140444; doi:10.3389/fnmol.2023.1154203)
Supplement: Supplementary file 1 [file Data_Sheet_1.PDF]

Mouse strain	Brain area	Mouse tag	Sex	Age (weeks)	Genotype
YACMJD84.2 and YACMJD15.4 transgenic mice	cerebellum	119.0.0	M	7	Q15
		119.0.1	M	7	Q15
		P307.3	M	7	Q15
		117.0.2	F	7	WT
		117.0.4	F	7	WT
		120.0.0	M	7	WT
		115.0.3	F	7	Q84
		115.0.4	F	7	Q84
		116.0.2,4	M	7	Q84
		293.0.4	F	8	Q84/Q84
		292.0.2	F	9	Q84/Q84
		88.0.3	M	7	Q84/Q84
		78.0.0	M	45	Q15
		78.0.3	M	45	Q15
		82.0.1	F	45	Q15
		29.0.0	F	43	WT
		76.0.4	M	46	WT
		54.0.1	M	47	WT
		88.0.1	M	45	Q84
		76.0.0	M	46	Q84
		76.0.3	M	46	Q84
		55.0.4	F	47	Q84/Q84
		1.0.2,3	M	47	Q84/Q84
		50.0.3	M	48	Q84/Q84
	brainstem	119.0.0	M	7	Q15
		119.0.1	M	7	Q15
		P307.3	M	7	Q15
		117.0.2	F	7	WT
		117.0.4	F	7	WT
		120.0.0	M	7	WT
		115.0.3	F	7	Q84
		115.0.4	F	7	Q84
		116.0.2,4	M	7	Q84
		293.0.4	F	8	Q84/Q84
		292.0.2	F	9	Q84/Q84
		88.0.3	M	7	Q84/Q84
		78.0.0	M	45	Q15
		78.0.3	M	45	Q15
		82.0.1	F	45	Q15
		88.0.2	M	45	WT
		76.0.4	M	46	WT
		54.0.1	M	47	WT
		88.0.1	M	45	Q84
		76.0.0	M	46	Q84
		76.0.3	M	46	Q84
		67.0.3	M	45	Q84/Q84
		1.0.2,3	M	47	Q84/Q84
		50.0.3	M	48	Q84/Q84
Atxn3 KO mice	cerebellum or brainstem	WT-10	M	7	WT
		WT-11	M	7	WT
		WT-12	M	7	WT
		131-0	F	7	KO
		131-3	F	7	KO
		135-2	M	7	KO
		WT-4	F	39	WT
		WT-5	F	39	WT
		WT-6	F	39	WT
		97-1	F	43	KO
		99-1	F	43	KO
		99-4	F	43	KO

Supplementary Table 1: Sex and age of the mice used in this study. WT, non-transgenic or non-genetically modified wild type littermate; Q15, hemizygous YACMJD15.4; Q84, hemizygous YACMJD84.2; Q84/Q84, homozygous YACMJD84.2; KO, *Atxn3* knockout
